# Identification and validation of m5c-related lncRNA risk model for ovarian cancer

**DOI:** 10.1186/s13048-023-01182-6

**Published:** 2023-05-15

**Authors:** Chong Wang, Chunxiao Zhang, Shimin Yang, Jiangdong Xiang, Dongmei Zhou, Xiaowei Xi

**Affiliations:** Department of Obstetrics and Gynecology, Shanghai General Hospital, Shanghai Jiao Tong University School of Medicine, Shanghai, China

## Abstract

Ovarian cancer (OC) is one of the common malignant tumors that seriously threaten women's health, and there is a lack of clinical prognostic predictors, while m5c and lncRNA have been shown to be predictive of multiple cancers, including OC. Therefore, our goal was to construct a risk model for OC based on m5c-related lncRNA.340 m5c-related lncRNA were identified and a novel risk model of OC ground on nine m5C-related lncRNA was constructed using LASSO-COX regression analysis. Kaplan–Meier analysis showed there was a significant difference in prognosis between risk groups. We established a nomogram which was a good predictor of overall survival. In addition, GSEA was enriched in multiple pathways and immune function analysis suggested that immune infiltration varies depending on the risk group. In vitro experiments show that AC005562.1, a key lncRNA of the risk model, is highly expressed in OC cells and promotes OC cell proliferation. Finally, we further explored the potential biological markers of m5c-related lncRNA in OC with WGCNA analysis and established a ceRNA network. In conclusion,we have developed a reliable m5c-related prediction model and performed systematic validation and exploration of various aspects. These results can be used for the assessment of OC prognosis and the discovery of novel biomarkers.

**Supplementary Information:**

The online version contains supplementary material available at 10.1186/s13048-023-01182-6.

## Introduction

OC is a common gynecological tumor whose incidence ranks third among the total number of gynecological tumors, after cervical cancer and endometrial cancer [1]. Results indicate that OC has the highest death rate and the worst prognosis of all gynecologic cancers. Surgical resection and chemotherapy of the tumor remain the most common treatments for OC [2]. According to the SEER database, the data shows that the rate of new cases of OC was 10.6 per 100,000 women per year. There is a circumstance that most OC is found to be advanced and chemotherapy resistance results in high relapse rates and a worse prognosis [3]. Therefore, there is a high necessity to better understand the molecular mechanisms of OC and the functional predictive features that may facilitate personalized survival prediction and provide the best treatment for patients.

The modification of RNA 5-methylcytosine (m5C) uses S-adenosyl-l-methionine as a donor methyl for the formation of m5C. The process of m5C methylation mainly includes the related methyltransferases, demethyltransferases, and the related binding proteins. The presence of RNA m5C modification has now been found in tRNA, snRNA, miRNA, lncRNA, and eRNA [4–7]. Many studies give evidence that methylation modifications act in promoting tumor metastasis, invasion, and drug resistance [8–10]. In addition, m5C methylation has been found to affect the survival risk associated with tumors such as OC [11], hepatocellular carcinoma [12], and low-grade glioma [13].

Long-stranded non-coding RNA (lncRNA), a type of non-coding RNA above 200 nucleotides in length., influences tumor development by inhibiting the growth of tumors through participating in tumor growth and metastasis [14], malignant transformation [15], and dynamic changes in the tumor microenvironment [16] and other processes. Studies show that in many malignancies, methylation-related genes have been proven to influence tumor progression through the regulation of lncRNA methylation levels, confirming that m5c modifications are widely present in lncRNA [17].

Both m5C-related genes and lncRNA are ideal diagnostic and prognostic markers. Increasing evidence suggests that m5C-related genes and lncRNA can predict prognosis in a variety of cancers with good predictive results. For instance, m5c-related genes have the ability to predict the prognostic value of lung squamous cell carcinoma patients [18]. For pancreatic ductal adenocarcinoma, the m5c-related lncRNA signature not only predicts prognosis independently but also provides accurate survival rate predictions [19]. Besides, m5c-related lncRNA signature stratify prognosis and response to chemotherapy in low-grade glioma patients [20]. Although m5C regulators and related lncRNA play an important role in the diagnostic and prognostic process, few studies have been conducted on the relationship between m5C-related lncRNA and OC.

The objective of this study is to develop and validate a risk model using m5c-related lncRNA. Furthermore, to reveal this prognostic model's potential functions, the relationship between signature and immune infiltration, and drug sensitivity are explored. Meanwhile, GSEA and WGCNA analysis studied the biological features as well as molecular pathways in patients with different prognoses of OC. Finally, the signature lncRNA, which is performed in vitro experiments, further illustrated the reliability of the model.

## Materials and methods

### Data extracted

The processed OC RNA sequencing profiles and related patient clinical characteristics were extracted from The Cancer Genome Atlas (TCGA)(http://portal.gdc.cancer.gov/). We choose the Transcripts Per kilobase Million (TPM) format for subsequent analyses. After excluding cases without survival information and secondary surgery, 375 OC samples were incorporated into our study. Table 1 provides detailed clinical data for OC patients. Clinical variables involved age, stage, grade, treatment, follow-up time, dimension, lymph nodes invasion (LNinvasion), vascular invasion (Vinvasion), status, and survival status.

**Table 1:** The detailed clinical characteristics of the OC patients

Characteristics	Unknow		Total set(*n* = 375)	Testing set (*n* = 187)	Training set (*n* = 188)	*P*
Age,n(%)	0	age ≤ 65	257(68.533)	122(65.241)	135(71.809)	0.171
		age > 65	118(31.467)	65(34.759)	53(28.191)	
Status,n(%)	47	Tumor free	84(25.610)	36(22.222)	48(28.916)	0.165
		With tumor	244(74.390)	126(77.778)	118(71.084)	
Vascular invasion,n(%)	272	No	40(38.835)	17(37.778)	23(39.655)	0.846
		Yes	63(61.165)	28(62.222)	35(60.345)	
Lymph nodes invasion,n(%)	228	No	47(31.973)	20(31.746)	27(32.143)	0.959
		Yes	100(68.027)	43(68.254)	57(67.857)	
Grade,n(%)	1	G1	1(0.267)	0(0.000)	1(0.535)	0.935
		G2	42(11.230)	22(11.765)	20(10.695)	
		G3	321(85.829)	160(85.561)	161(86.096)	
		G4	1(0.267)	0(0.000)	1(0.535)	
		GX	9(2.406)	5(2.674)	4(2.139)	
Stage,n(%)	3	Stage I	1(0.269)	1(0.535)	0(0.000)	0.716
		Stage II	21(5.645)	12(6.417)	9(4.865)	
		Stage III	292(78.495)	147(78.610)	145(78.378)	
		Stage IV	58(15.591)	27(14.439)	31(16.757)	
Dimension, mean(± SD)	14	(cm)	0.905 ± 0.394	0.876 ± 0.327	0.934 ± 0.450	0.158

### Identification of m5c-related lncRNA

The correlation between m5c regulators and lncRNA was used to identify m5c-related lncRNA, which was selected under the rule that |correlation coefficient|> 0.4, *P* < 0.001. We used the R package “limma” to output the m5c-related lncRNA expression matrix. At the same time, an alluvial diagram was plotted to depict the relationship between the regulators and lncRNA.

### Construction of the risk model and verify the signature

The matrix was analyzed by univariate cox regression and the 14 lncRNA that related to prognosis were obatained. Then, the entire set classified into the training set (*N* = 188) and testing set (*N* = 187) by R language randomly. Nine m5c-related lncRNA in the training set were identified by using the LASSO-COX regression analysis, and a prognostic model was builted. The risk score's calculation formula was set as RiskScore = ∑EXPi × COEi, in which EXP meant the expression value of the lncRNA, and COE referred to the regression coefficient. Based on the median risk score, patients in the training set were split into high-risk and low-risk groups. The Kaplan–Meier and ROC curves were made by using the "Survival" and "SurvivalROC" packages, respectively, while the PCA scatter plot was created by using the "ggplot2" tool. Meanwhile, the above analysis of the testing set has also been made accordingly. Kaplan–Meier analysis was used to explore the relationship between risk scores and different clinical subgroups.

### Construct the nomogram

Univariate and multivariate Cox regression analyses were undertaken to determine if the prognostic signature might be independent of other clinical characteristics. Depending on the risk score and other independent clinical prognostications, an OC patient prognostic nomogram was created by the “rms” package to anticipate the likelihood of 1-, 3-, and 5-year OS. Calibration plots were used to compare predicted survival with actual survival. ROC curve were used to analyze the sensitivity and specificity of the nomogram.

### GSEA

Gene function was analyzed by using GSEA version 4.1.0 software from the MSIGDB database on the GSEA website (http://software.broadinstitute.org/gsea/msigdb) and the standard weighted enrichment approach was used for enrichment analysis. In this study, one thousand times were specified for the random combination. Hallmark, GO and KEGG enrichment analyses were executed using the GSEA analysis for the high and low-risk groups. FDR q-val < 0.25, |NES|> 1, and NOM *p*-val < 0.05 were regarded as significant enrichment parameters.

### Estimation of Tumor-Infiltrating Immune Cells and ssGSEA

We calculated the enrichment levels for 29 immune gene sets per OC sample by using the single-sample gene-set enrichment analysis (ssGSEA) score. Meanwhile, within each sample, the proportion of 22 different types of immune cells was calculated using the CIBERSORT R package.

### Prediction of chemotherapy response

To predict chemotherapy responses for each patient based on the ovarian cell lines gene expression matrix and drug sensitivity data from Genomics of Drug Sensitivity in Cancer (GDSC). Each sample’s sensitivity to chemotherapeutic drugs was estimated by the R package “pRRophetic” which computed the half maximal inhibitory concentration (IC50) for each patient through ridge regression.

### CeRNA network construction

WGCNA is a comprehensive weighted association network analysis software based on the R language. This study performed WGCNA analysis on the lncRNA of different risk groups. The WGCNA in R software was going into performing this process and visualizing it. After checking the missing values and identifying outliers, the minimum soft threshold whose scale-free topology fitting index reaches 0.9 was calculated to construct hierarchical clustering that co-expresses the network and module identification. Pearson correlation analysis was dedicated to calculating the modules most related to risk. In the related module, the significant GS of the gene and the MM of the module member were going by identifying the highly related genes, and we set MM > 0.65, and GS > 0.2. Based on the lncRNA we get from WGCNA, we use Lncbase v3 to predict the lncRNA–miRNA relationships. After that, we use miRDB to predict the miRNA–mRNA relationships. The ceRNA network graph was visualized by Cytoscape v3.6.0 and mRNA was imported into STRING(https://string-db.org/) to draw the PPI network.

### In vitro* assays*

The cell lines SKOV-3, A2780, HEY, and IOSE 80 were obtained from the National Collection of Authenticated Cell Cultures (Shanghai, China). siRNA against human AC005562.1 were synthesized by GenePharma (Shanghai, China), and transfected into cells using Lipofectamine 3000 (Invitrogen, Carlsbad, CA, USA). Total RNA has been extracted from cells using TRIzol Reagent(Invitrogen, Carlsbad, CA, USA). According to the manufacturer’s instructions for the Reverse Transcription Kit (EnzyArtisan, China), RNA was reversely transcribed into cDNA. Using cDNA as a template, 2 × S6 Universal SYBR qPCR Mix (EnzyArtisan, China) and quaint studio 7 flex real-time PCR system (ThermoFisher) were going to detect real-time Quantitative PCR(rt-qPCR).

The transfected OC cells were seeded in 96-well plates, and cell proliferation was measured using cell counting kit-8(CCK-8)(Dojindo, Tokyo, Japan). In addition, the AC005562.1 primers were the following: AC005562.1 -F, 5′- tggtcgtcatggaccggaag -3′; AC005562.1 -R: 5′- cttgcgagccaaaagtcctc -3′.

### Statistical analysis

Statistical analysis was conducted by R software (version 4.0.3), Perl software (version 5.3), and Graphpad Prism 9.3.0. Univariate and multivariate Cox proportional hazard models, LASSO method, Kaplan–Meier method, PCA, and ROC analysis were used in this study. Moreover, the rt-qPCR results were quantified by the ΔΔCT method and analyzed using the Student’s t-test.

## Results

### Identification of m5c-related lncRNA

**Fig. 1:**
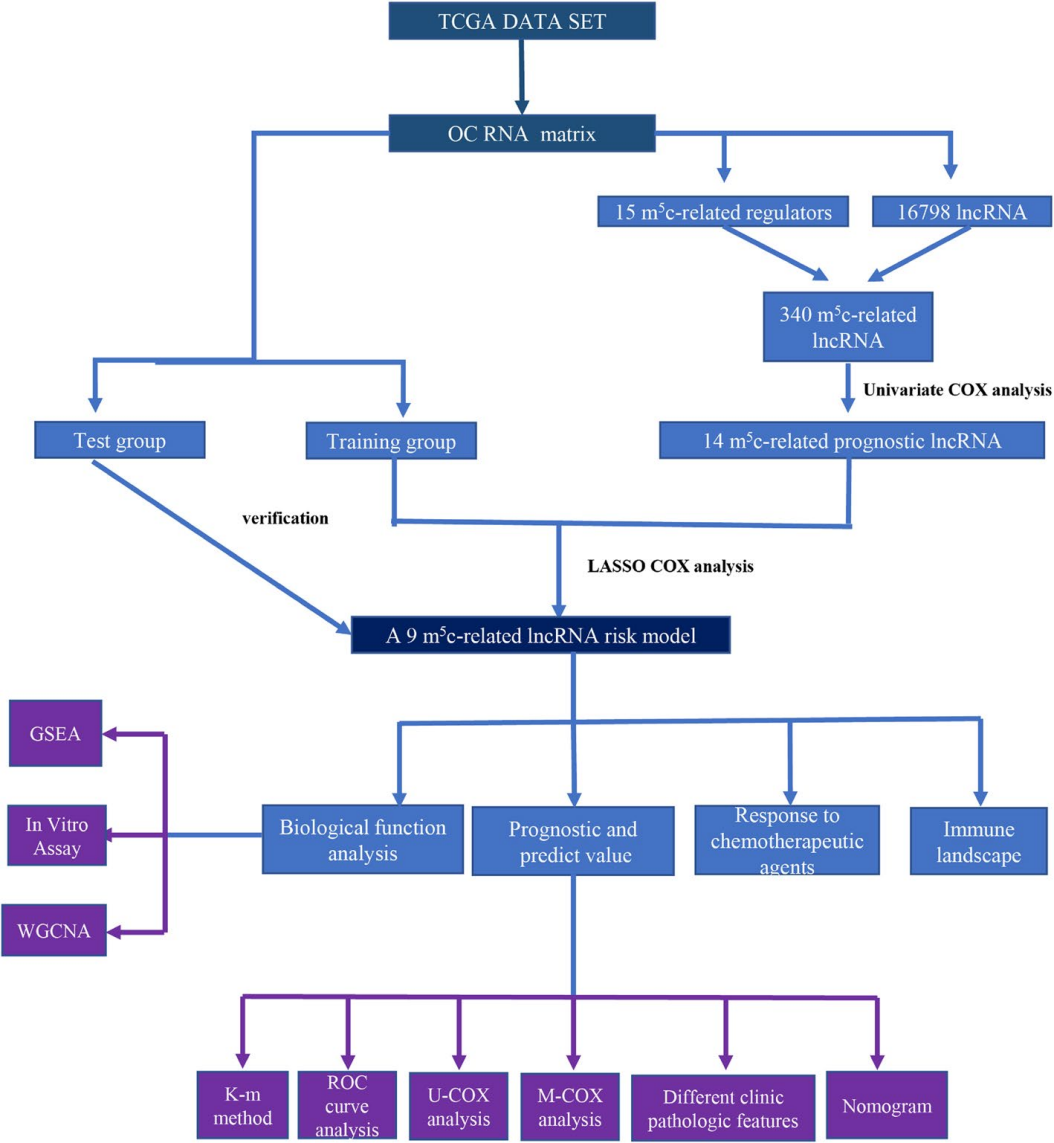
Flow chart of this study

**Fig. 2:**
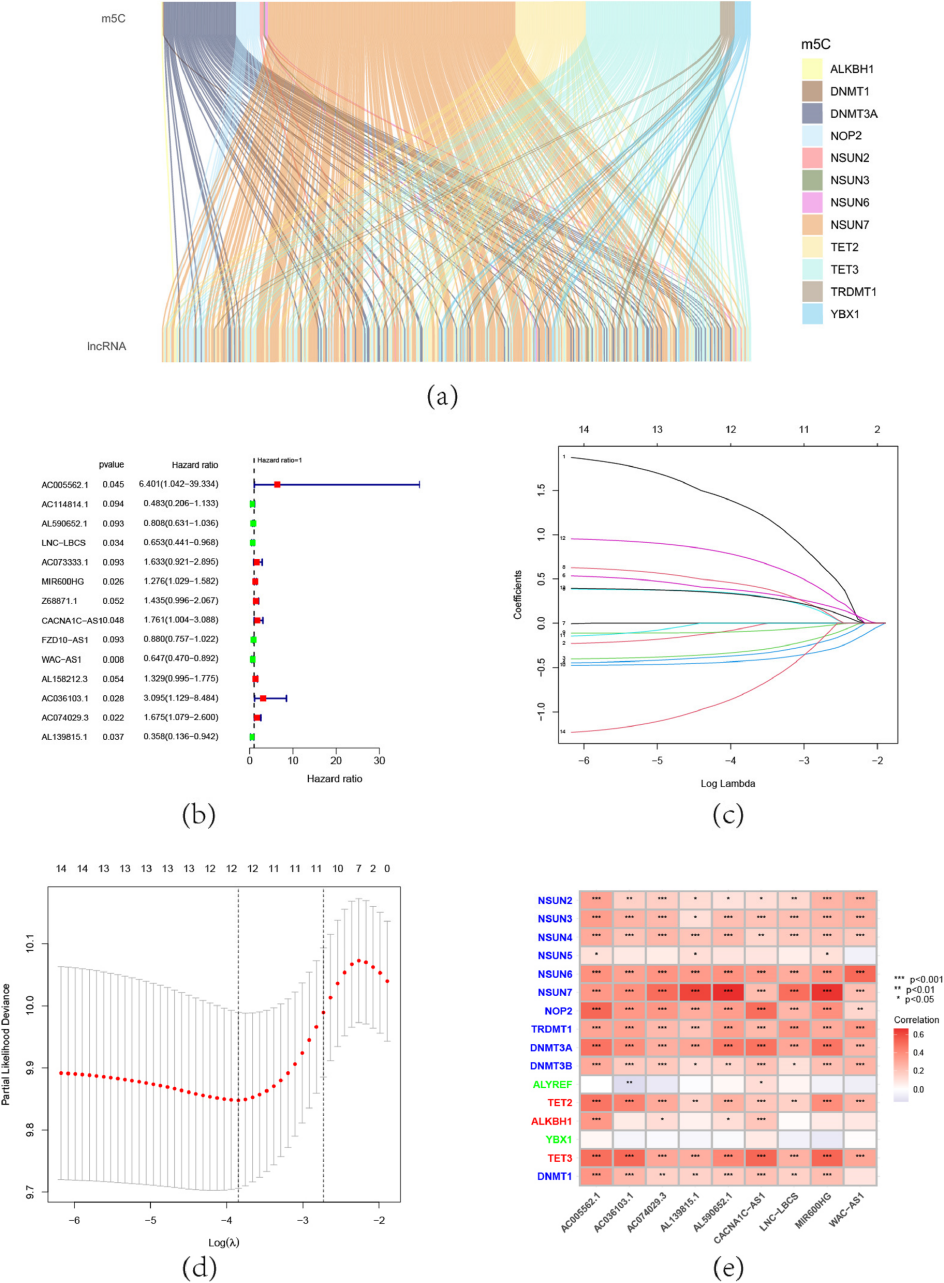
Construction of the risk model. **a** An alluvial diagram which plotted to depict the relationship between the regulators and lncRNA. **b** 14 prognosis-associated lncRNA were obtained by univariate cox regression analyses, show their hazard ration with a forest diagram. **c** LASSO analysis of 14 prognosis-associated lncRNA. **d** Cross-validation curve for adjusting parameter selection in LASSO analysis. **e** The correlation graph illustrate that risk model-related lncRNA has good correlation with m5c regulators

In order to visualize this study, a flow chart (Fig. 1) was provided which illustrated the framework of the research. We obtained 15 m5c regulators from the published articles. The list of related gene names was provided in Table S1. By referring to the rule of identification, 340 m5c-related lncRNA were identified. The relationship between the m5c regulators and lncRNA was depicted through an alluvial diagram (Fig. 2a).

### Establishment of the Risk Model

**Fig. 3:**
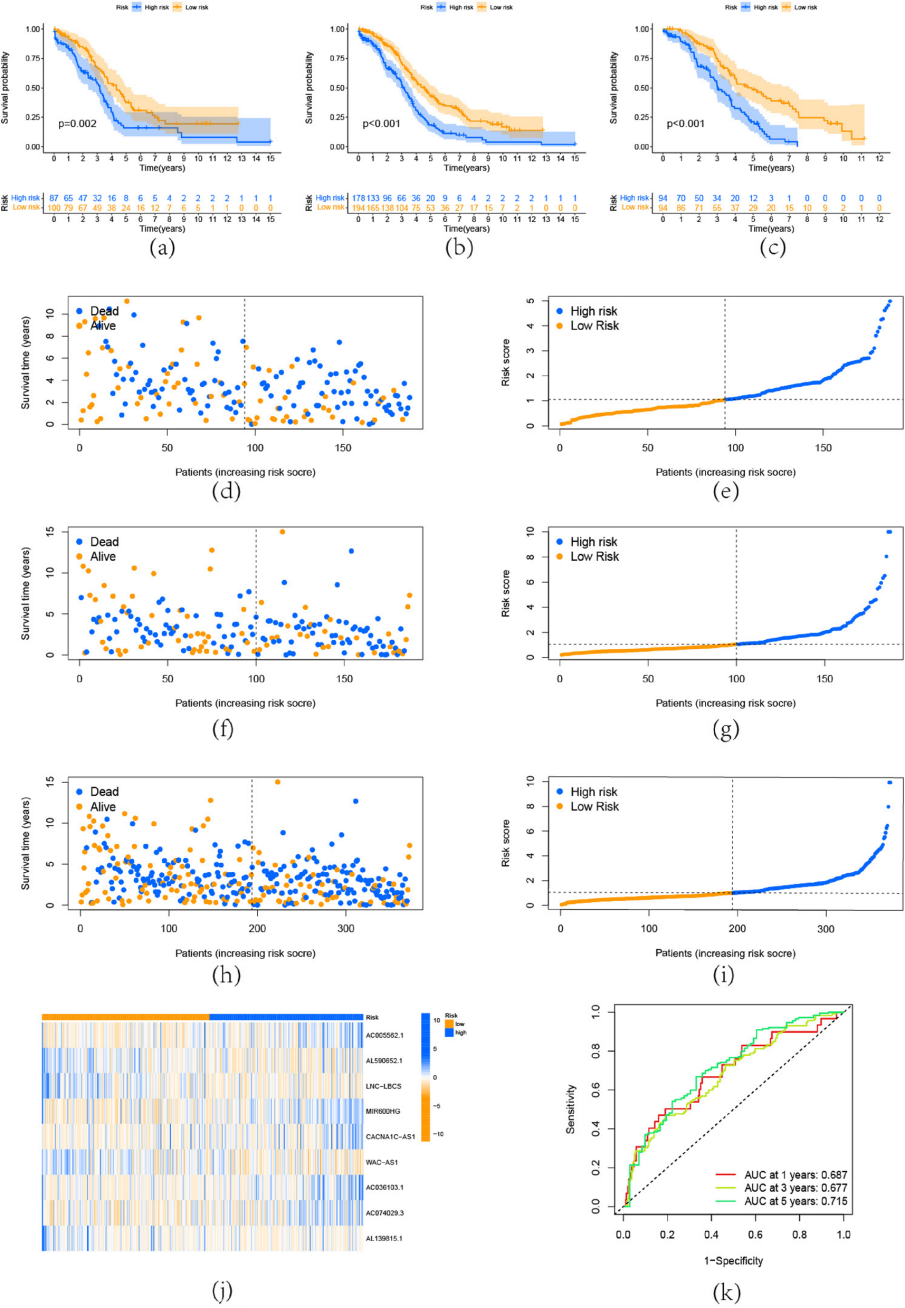
Prognostic value of the risk model. **a**-**c** Kaplan–Meier(KM) curve analysis of the training, testing, and entire set, in each group, the prognosis was better in the low-risk group than in the high-risk group. **d**,**f**,**h** The survival status for each OC patient in the training, testing, and entire set. **e**,**g**, **i** Risk score distribution for each OC patient in the training, testing, and entire set. **j** Model-related lncRNA expression heatmap. **k** The ROC curve, AUC at 1,3,5 year are 0.69, 0.68, and 0.72

By using the univariate cox regression, 14 prognosis-associated lncRNA were found (Fig. 2b)0.375 OC samples were separated into a training set (*N* = 188) and a testing set (*N* = 187). This m5C-related lncRNA underwent the LASSO regression and multivariate Cox proportional hazard regression analyses in order to further investigate the prognostic significance of this lncRNA in OC and to help choose a reliable risk model for prognosis prediction. Specifically, we further screened the lncRNA by lasso regression (Fig.  2c), we select the lambda corresponding to the left dashed line in the cross-validation curve (Fig. 2d) to obtain the best model and 12 prognosis-associated lncRNA were found. After that, the 12 lncRNA that related to prognosis were subjected to multivariate Cox regression analysis. As a result, it was able to identify 9 lncRNA, and the corresponding coefficient values are shown in Table 2. The correlation graph illustrates the relationship between these lncRNA and m5c regulators (Fig. 2e). The risk score formula was as follows: RiskScore = (2.097107) * AC005562.1 + (1.016992) * AC036103.1 + (0.355) * AC074029.3 + (-1.21116) * AL139815.1 + (-0.47768) * AL590652.1 + (0.618166) * CACNA1C-AS1 + (-0.43103) * LNC-LBCS + (0.479378) * MIR600HG + (-0.51962) * WAC-AS1. The risk score of each patient was calculated by the formula and the patients were divided into high- and low risk- groups according to the median risk score of the training set. After grouping, there were 94 high-risk and 94 low-risk patients in the training set while there were 87 high-risk and 100 low-risk in the testing set. In the training and testing set, it was discovered that high-risk patients were associated with more fatalities (Fig. 3a,3b). The survival status (Fig. 3d,3f) and risk score distribution (Fig. 3e,3g) for each OC patient in the training and testing were shown, it implies that the high-risk group experiences a greater mortality rate and a shorter survival period. We performed the above analysis on the entire dataset to complete the internal validation and obtained the same results (Fig. 3c,3h,3i). Heatmap was used to represent model-related lncRNA expression (Fig. 3j). The areas under the curve (AUC) at 1,3,5 year are 0.69, 0.68, and 0.72 (Fig. 3k), demonstrating the risk model's proficiency in OC prognostic prediction.

**Table 2:** The HR values and coefficient values of risk model-related lncRNA

id	HR	HR.95L	HR.95H	pvalue	coefficient
AC005562.1	6.400524	1.041513	39.33384	0.045084	2.097107
AC036103.1	3.095322	1.129352	8.483647	0.028061	1.016992
AC074029.3	1.674854	1.078993	2.599771	0.021513	0.355
AL139815.1	0.357883	0.136028	0.941573	0.037348	-1.21116
AL590652.1	0.808167	0.630557	1.035804	0.092543	-0.47768
CACNA1C-AS1	1.761052	1.004222	3.088267	0.048306	0.618166
LNC-LBCS	0.653078	0.440592	0.96804	0.033861	-0.43103
MIR600HG	1.275899	1.029058	1.581949	0.026346	0.479378
WAC-AS1	0.647499	0.470059	0.891919	0.007815	-0.51962

### Prognostic and Predict Value of Risk Modle

**Fig. 4:**
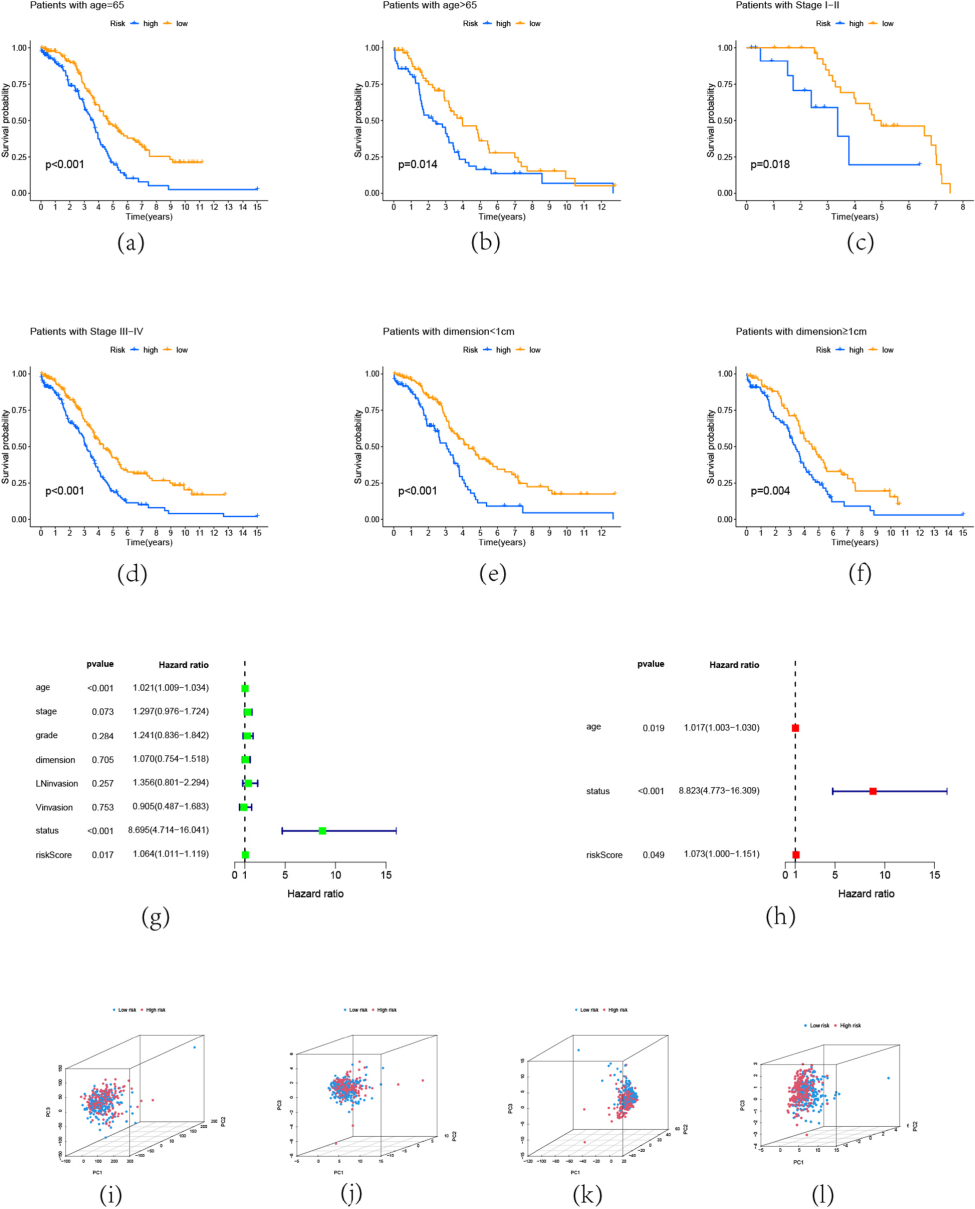
Prognostic and Predict Value of Risk Modle. Patient OS K-M curves, categorized by (**a**,**b**) age, (**c**,**d**) stage, and (**e**,**f**) dimension. All clinical subtypes showed significant differences (*p* < 0.05).The risk model has good predictive power in different situations. OS of OC patients: univariate and multivariate analysis in entire set (**g**, **h**) to determine whether the risk score is a separate risk factor. Comparison of PCA between two groups in entire set using (**i**) all genes, (**j**) m5c gene, (**k**) m5C-related lncRNA, and (**l**) risk model-related lncRNA

A subgroup study proceeded to determine if the risk score could be used in other situations. It was found that the risk score predicted overall patient survival in a variety of conditions (Fig. 4a-4f). These results show how well the risk model is at predicting future events.

Then, to determine more about the predictors of OC, univariate and multivariate Cox regression analyses were conducted in the entire set. Age and risk score had an impact on the prognosis of the OC patients in the training cohort, as shown by the findings of the univariate Cox regression analysis. (Fig. 4g). Furthermore, the multivariate Cox regression analysis revealed that the risk score remained significantly connected with the prognosis of OC patients. (Fig. 4h). This led to the conclusion that risk score is a separate risk factor.PCA results also showed distinct between the two groups in the entire set based on all genes, m5c gene, m5c-related lncRNA, and risk model-related lncRNA (Fig. 4i-l). The high-risk group is more distinct from the low-risk group in accordance with the risk-related lncRNA.

### Nomogram

**Fig. 5:**
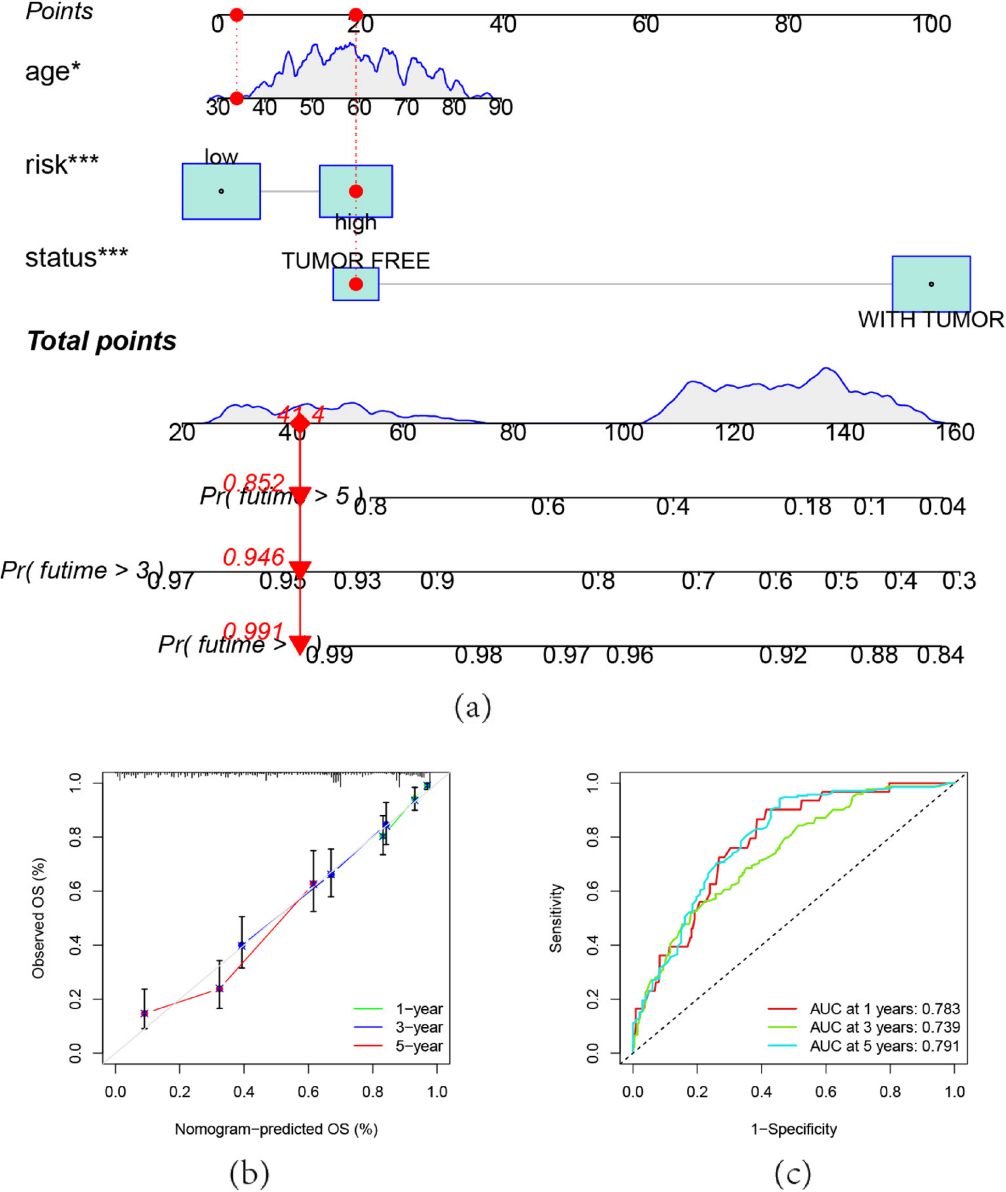
Nomogram. **a** The Nomogram model to forecast the likelihood that OC patients would survive at 1, 3, and 5 years. **b** The Nomogram model relevant calibration curves, the gray diagonal line indicates the ideal column line diagram, the green and blue red lines represent the predicted 1-year, 3-year, and 5-year overall survival of the patients, respectively. **c **The ROC curve for the nomogram, the AUC at 1,3,5 year are 0.78, 0.74, and 0.79

Place clinical variables like status, age, and risk into the nomogram model to forecast the likelihood that OC patients would survive at 1, 3, and 5 years. (Fig. 5a). It was demonstrated that the relevant calibration curves would make a recent prediction of the findings at 1, 3, and 5 years (Fig. 5b). As a result, the nomogram that included clinical characteristics and risk was reliable and accurate, and it could be used in predicting the OC patients' 5-year OS rate. The AUC of ROC curve for the nomogram at 1,3,5 year are 0.78, 0.74, and 0.79, demonstrating the predictive effect of nomogram.

### Gene Set Enrichment Analysis (GSEA)

**Fig. 6:**
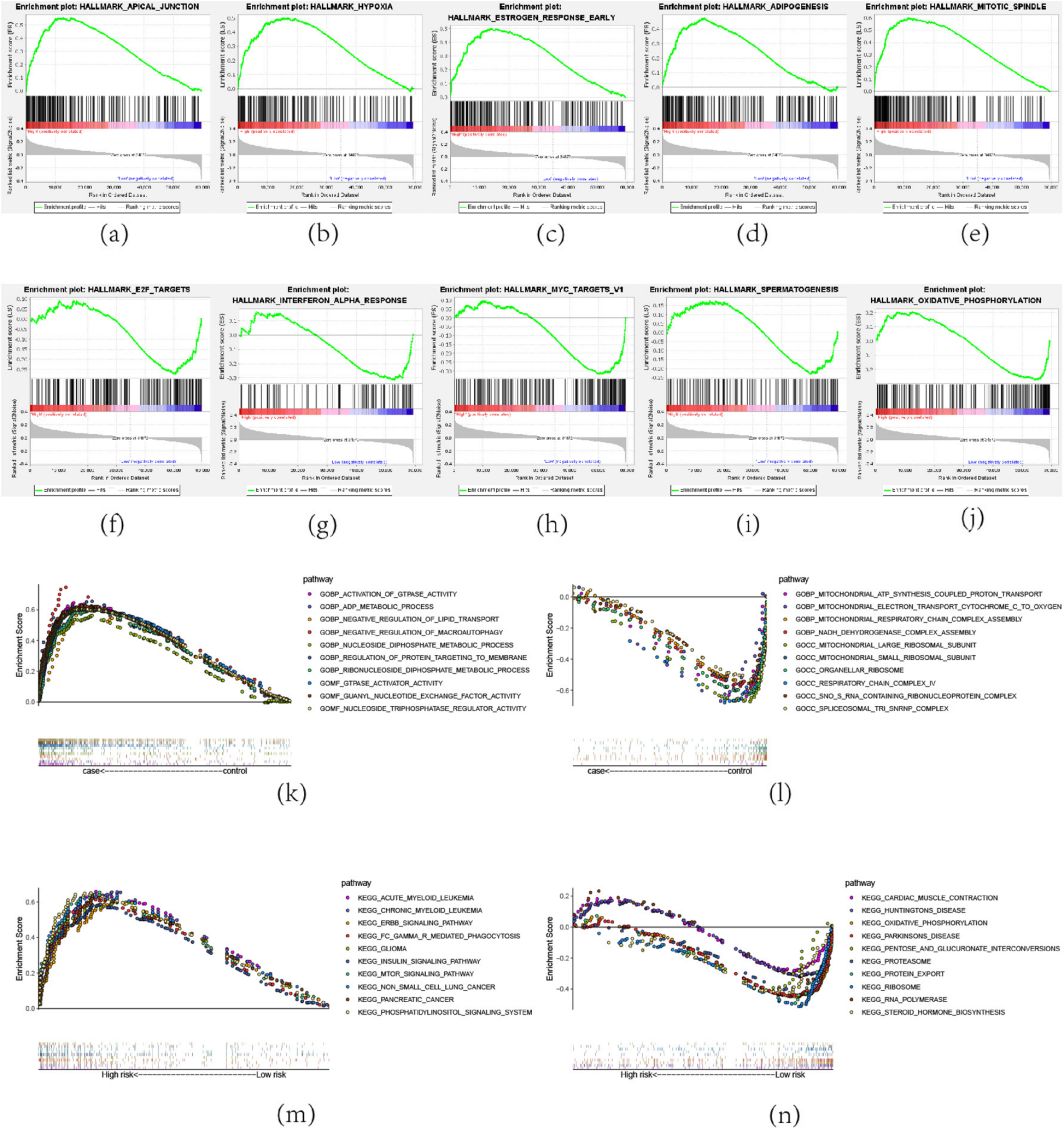
GSEA. The top five hallmark pathways that are more prevalent in the high-risk group (**a**-**e**) and low-risk group (**f**-**j**). The top ten GO enriched in the high-risk group (**k**) and low-risk group (**l**). The top ten KEGG pathways enriched in the high-risk group (**m**) and low-risk group (**n**)

To investigate the functional characteristics of the risk model, this study performed the hallmark pathway, GO enrichment, and KEGG pathway analyses between the two risk subgroups by the GSEA. The top five hallmark pathways that are more prevalent in the high-risk group (Fig. 6a-e) include apical junction, hypoxia, estrogen response early, adipogenesis, and mitotic spindle, while e2f targets, interferon alpha response, MYC targets v1, spermatogenesis, and oxidative phosphorylation enriched in the low-risk group (Fig. 6f-j). Go analysis showed that the DEGs enriched in multiple GO gene sets including Biological Process (BP), Cellular Component (CC), and Molecular Function (MF), such as activation of GTPase activity, mitochondrial large ribosomal subunit, GTPase activator activity, and so on(Fig. 6k-l). KEGG analysis also significantly enriched the ERBB signaling pathway, insulin signaling pathway, and MTOR signaling pathway, among others(Fig. 6m-n). These findings demonstrated that distinct signaling pathways exist between the two groups, which may help to explain why there was a significant variation in prognosis between groupings.

**Fig. 7:**
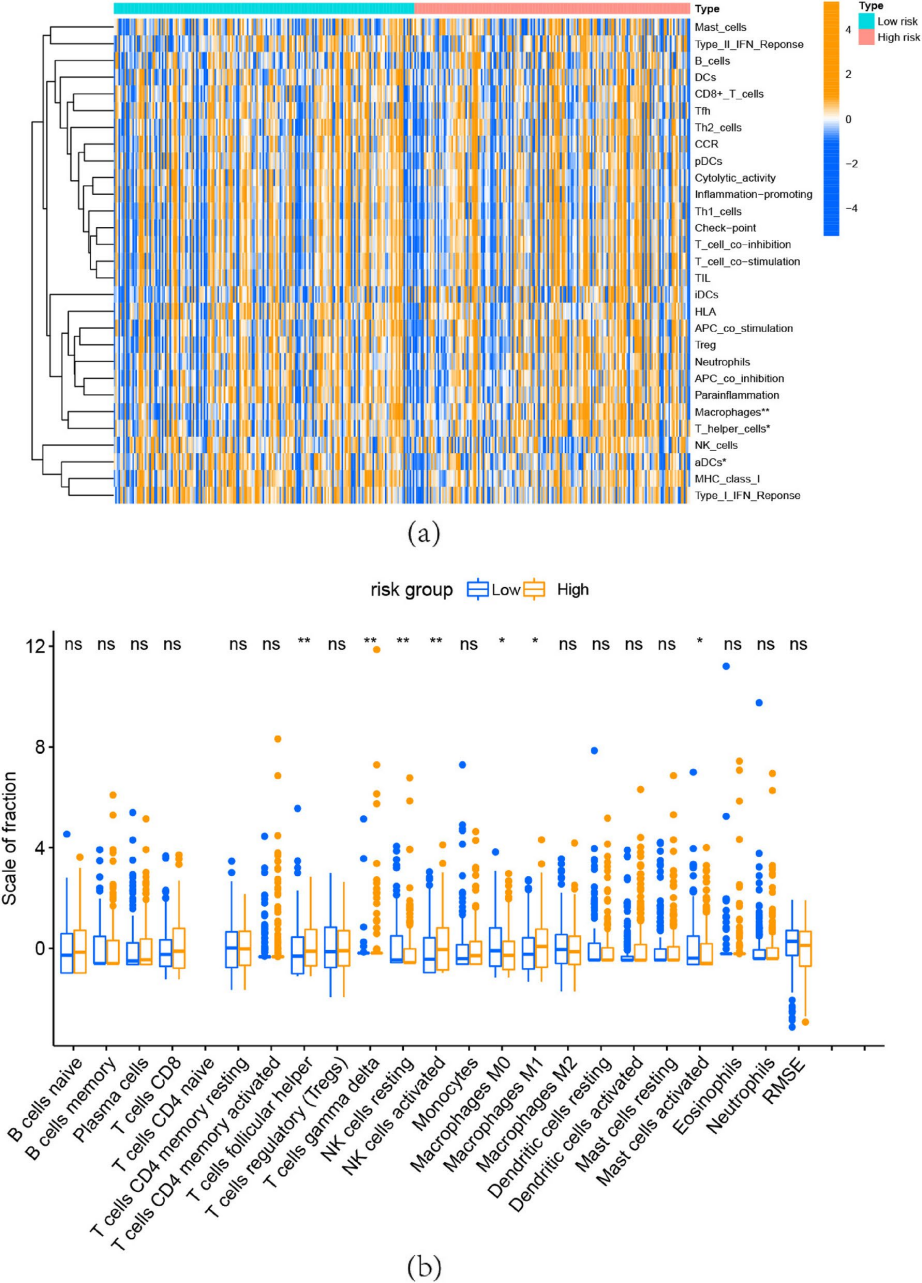
Estimation of Tumor-Infiltrating Immune Cells and ssGSEA (**a**) ssGSEA analysis for 29 immune gene sets. *p < 0.05, **p < 0.01 (**b**) The scale of 22 different immune cell infiltration scores calculated by CIBERSORT in the two groups. (ns) Non-significant, *p < 0.05, **p < 0.01, and ***p < 0.001

Correlation Between Immune Landscape and Risk Model.ssGSEA analysis showed that ADCs, T helper cells, and macrophages differed between the two groups while the others did not (Fig. 7a). The scale of 22 different immune cell infiltration scores in the two groups was shown in a boxplot.The high-risk group was closely correlated with T cells follicular helper, T cells gamma delta, NK cells activated, and Macrophages M1, otherwise, showed a decreased infiltration of NK cells resting, Macrophages M0, and Mast cells activated (Fig. 7b), which confirmed significant differences in immune infiltration across risk subgroups

### Response to chemotherapeutic drugs

**Fig. 8:**
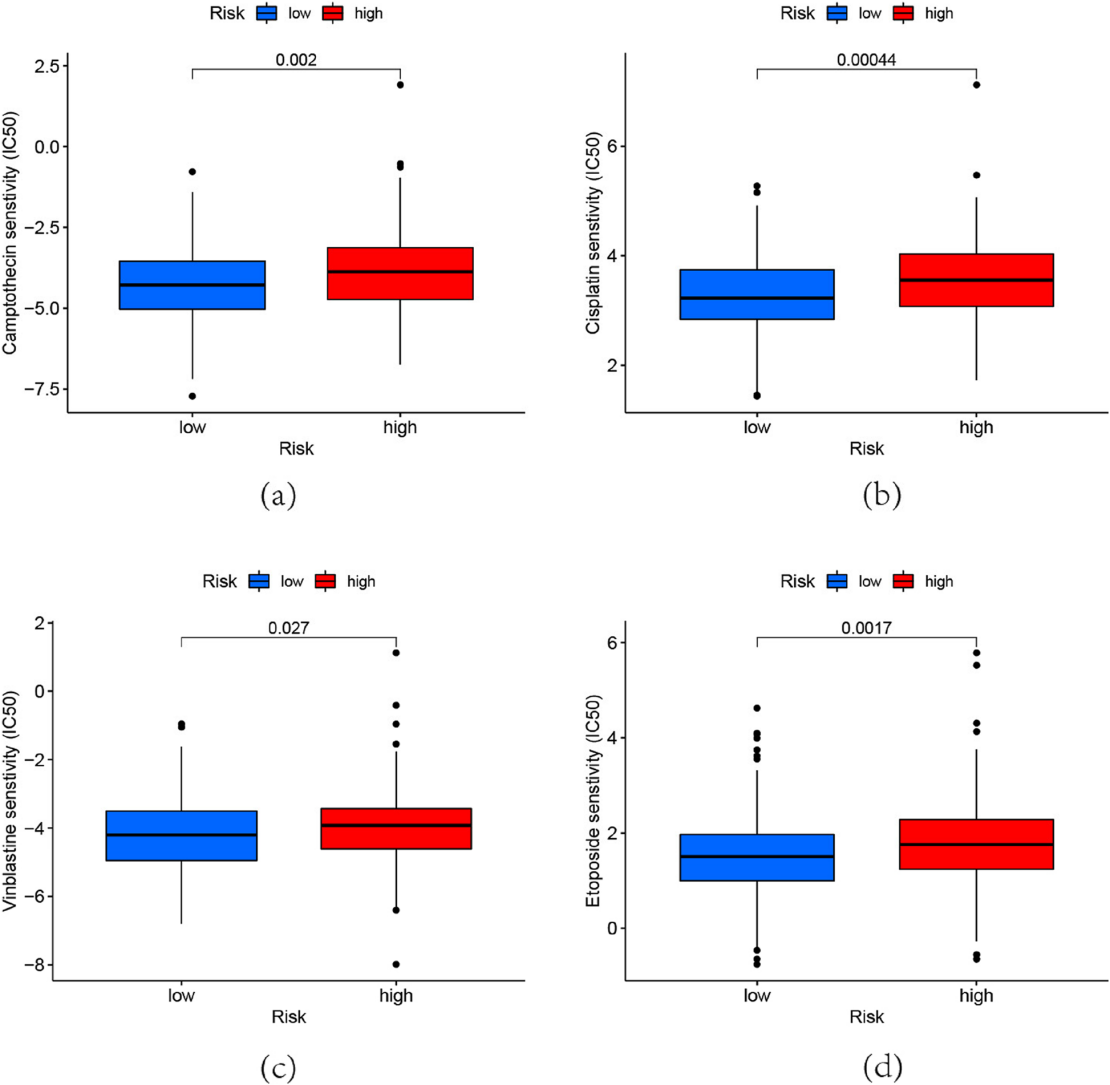
Patients’ sensitivity to OC chemotherapeutic agents, calculated by the R package “pRRophetic”. **a** Camptothecin. (*p* = 0.002) (**b**) Cisplatin. (*p* = 0.00044) (**c**) Etoposide. (*p* = 0.027) (**d**) vinblastine. (*p* = 0.0017)

We assessed how OC patients with various risk scores responded to 137 chemotherapy agents, of which 49 had significantly different sensitivities. In particular, patients’ sensitivity to camptothecin, cisplatin, etoposide, and vinblastine was higher in the low-risk group, which were commonly OC chemotherapeutic agents(Fig. 8a-d). Resistance to these drugs might be related to the survival risk in the high-risk group. In the high-risk group, we discovered that 41 drugs had lower IC50 values(Figure S1), providing a reference for the selection of chemotherapeutic agents in clinical practice.

### CeRNA network construction

**Fig. 9:**
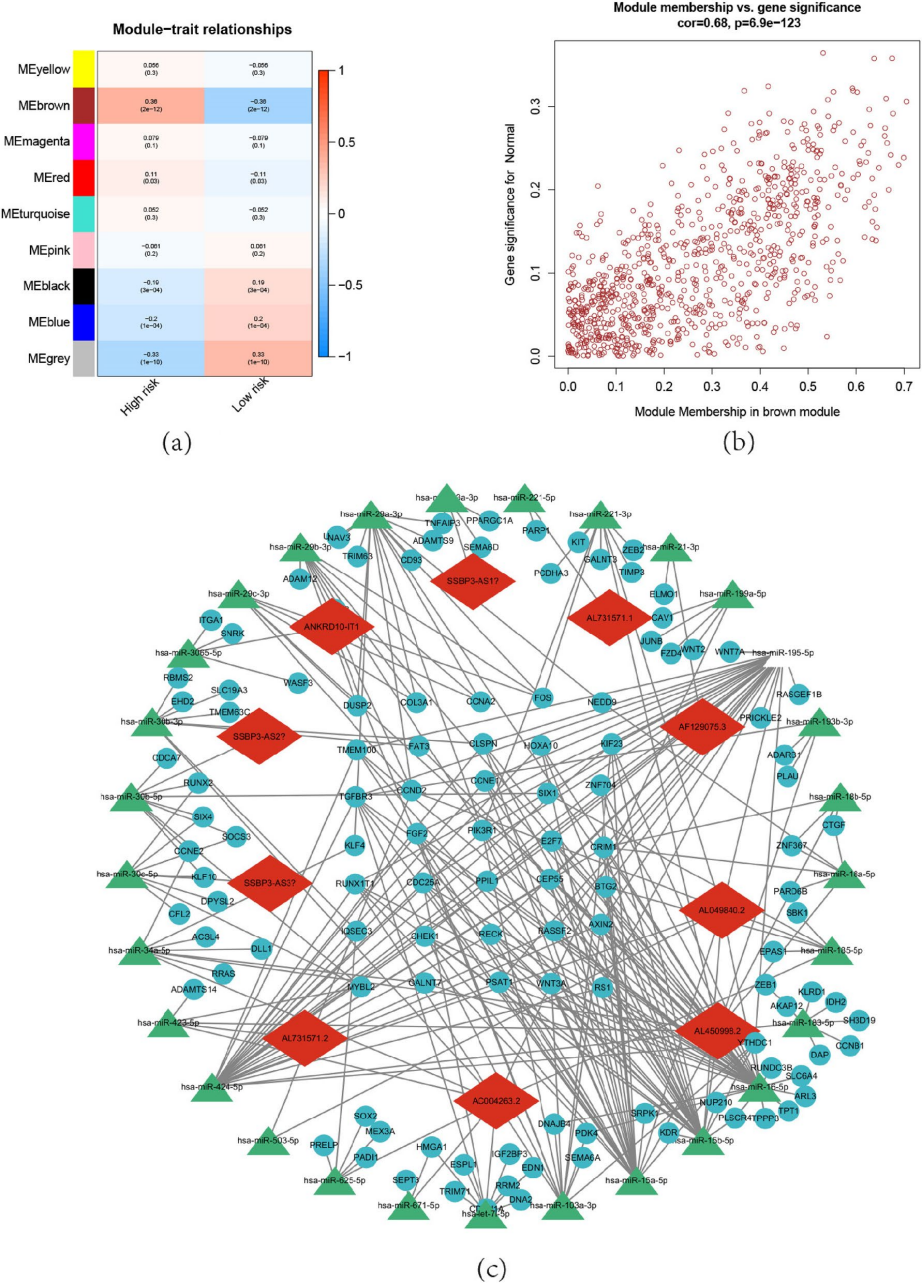
WGCNA and ceRNA network. **a** The relationship between risk subgroups and the module eigengenes, intensity and direction of correlations are indicated on the right side of the heatmap (red, positively correlated; green, negative correlated. **b **The module membership in the brown module. **c** The ceRNA network demonstrate the relationship between model-related lncRNA and miRNA-mRNA

To further investigate the role played by m5c-related lncRNA in the construction of the prognostic model of OC, we created a ceRNA network using the WGCNA method and used PPI analysis to demonstrate the interactions between the relevant mRNA-expressed proteins. The brown module shows an extremely high correlation with risk (Fig. 9a,b). Selected lncRNA within the module and predicted miRNA sponged by lncRNAs. miRDB for predicting miRNA-mRNA relationships. We show the specific regulation mechanism through the ceRNA network ((Fig. 9c). The PPI network demonstrated the role of the target mRNA (Figure S2a). GO analysis revealed targeted mRNA enriched in several functions and process (Figure S2b,c).

### In Vitro experiments

By using rt-qPCR, it was determined that the three OC cell lines had much greater levels of AC005562.1 expression than normal ovarian epithelial cells (Figure S3a). siRNA knockdown efficiency was also verified by rt-qPCR (Figure S3b,c). As shown in the Figure (Figure S3b,c), we chose to select the two combinations with the highest knockdown efficiency for cell proliferation assays separately. The CCK-8 results demonstrated that lncRNA knockdown prevented OC cell proliferation (Figure S3d,e).

## Discussion

OC is a high-mortality gynecologic malignancy [21], and effective prognostic assessment methods are beneficial for the timely identification of patients. The prognostic predictive power of m5c-related lncRNAs has been demonstrated in many tumors, but there are limited reports in OC. The purpose of this study was to develop an m5c lncRNA prognostic model for OC.

In this study, we identified 340 m5c-related lncRNA in the TCGA dataset, and 9 lncRNAs were identified and prognostic modeled by lasso regression and multifactorial cox analysis. A novel prognostic signature of m5c-related lncRNA could precisely distinguish the OS of OC patients. The training and validation set tested the classification ability of the risk model. The m5C methylation affects the survival risk associated with many tumors, and in hepatocellular carcinoma, high expression of NSUN4 was significantly associated with survival outcomes [22], NSUN4 was also associated with increased risk of breast, ovarian, and prostate cancers [23]. The expression of NSUN2 is upregulated in major gynecologic neoplastic diseases [24], it is also elevated in breast cancer and head and neck neoplasms [25, 26]. Risk scores based on m5c-related lncRNA characteristics were determined to be an independent predictor when controlling for clinical variables by univariate and multivariate regression. Moreover, the prognostic model has good predictive power of OS in subgroups of patients with different clinical features.

We also created a nomogram where the observed rates for the 1-, 3-, and 5-year operating systems show perfect agreement with the predicted rates in the correlation chart. This nomogram will provide a reference for clinicians to assess the prognosis of OC patients.

It has been demonstrated that lncRNA is crucial to the development and progression of OC. 9 m5c-related lncRNA were obtained in our study, and in vitro assay proved lncRNA AC005562.1 function in OC cells. All but AC074029.3 and AL139815.1 of prognostic m5c-related lncRNA have been studied in cancer. WAC-AS1 was shown to promote glycolytic efficiency and proliferation in hepatocellular carcinoma cells [27]. It was also included in another predictive model for OS in OC patients [28]. Lnc-LBCS serves a tumor-suppressive effect in bladder cancer stem cells, which is tightly related to prognosis, treatment response, and clinical stage [29]. Expression of CACNA1C is indirectly affected by the hemimethylated of CACNA1C-AS1 CPG codon [30], which is considered the master gene of intestinal-type adenocarcinomas [31]. Interestingly, Zhu et al. used AL590652.1 [32] as one of the necroptosis-related lncRNA signatures in OC patients while AC036103.1 [33] is actively engaged in creating risk signatures for gastric adenocarcinoma. MIR600HG acts as an anticancer agent by inhibiting colorectal cancer cell stemness [34]. However, Liu et al. [35] found a carcinogenic role for MIR600HG in the development of oral squamous cell carcinoma cells. Therefore, the precise mechanisms of this lncRNA in cancer need to be further explored.

What ‘s more, GSEA analysis further revealed the biological functions that may be involved in risk model. Hypoxia was enriched in the high-risk group through HALLMARK analysis, while oxidative phosphorylation was enriched in the low-risk group. Metabolic is essential for cancer cell growth, survival, and proliferation [36]. In OC cells, an enhanced glycolytic phenotype was observed [37, 38], and PI3K/AKT, Myc, or hypoxia-inducible factor (HIF) was found to be involved in the glycolytic process [39]. In GO and KEGG analysis, they also enriched in glycolytic pathways such as KEGG INSULIN SIGNALING PATHWAY, GOBP ADP METABOLIC PROCESS. Focusing on the glycolytic may assist the management of OC. OC has been shown to possess immunogenicity [40], and immunotherapy for OC is receiving increasing attention [41]. An increase in immune infiltration was observed in the high-risk group, which would lead to a negative prognosis. The high recurrence rate of OC is frequently linked to chemotherapy resistance [42], which is one of the main causes of the poor survival rate of OC [43]. By predicting sensitivity to chemotherapeutic agents, we found that some OC chemotherapeutic agents were resistant in the high-risk group, this might be a predictive risk factor for individuals in the high-risk group. Additionally, we discovered that patients in the high-risk group were more sensitive to 41 drugs, and these findings will direct how clinical pharmaceuticals are used. WGCNA analysis further explored the possible mechanism and role of m5c-related lncRNA in OC, and the new biomarkers identified could be used for future studies.

However, there are some limitations of the study. In the beginning, we used only the TCGA dataset as a single source of data, lacking additional cohorts to validate the results. Secondly, although we experimentally validated the model with the highest coefficients of lncRNA, the confirmation of our results is required by more comprehensive in vivo and in vitro experiments. Last but not least, it’s necessary to evaluate the prognostic features in a real-world setting.

## Conclusions

In conclusion, we have developed a reliable m5c-related prediction model and performed systematic validation and exploration of various aspects. These results can be used for the assessment of OC prognosis and the discovery of novel biomarkers.

## Supplementary Information

**Additional file 1.** **Table S1.** The list of m5c regulators. **Figure S1 41**. drugs had lower IC50 values in the high-risk group. **Figure S2.** The PPI network and GO analysis. (a) The PPI network. (b) Biological process analysis. (c) Cellular component analysis and molecular function analysis. **Figure S3.** In vitro experiments. (a)AC005562.1 are overexpressed in OC cell lines. (b) siRNA-3 significantly knocked down the AC005562.1 in A2780. (c) siRNA-2 significantly knocked down the AC005562.1 in SKOV3. (d,e) OC cell viability was evaluated with CCK-8 assays at 0, 24, 48, and 72 h post-transfection. *P < 0.05, **P < 0.01, ***P < 0.001.

## Data Availability

TCGA(http://portal.gdc.cancer.gov/).GDSC (https://www.cancerrxgene.org/). The rest of the public databases used are indicated in the text.
