# Supplementary material for: Identification and validation of m5c-related lncRNA risk model for ovarian cancer
Source: J Ovarian Res. 2023 May 15;16:96. doi: 10.1186/s13048-023-01182-6 (PMC10184408; doi:10.1186/s13048-023-01182-6)
Supplement: Supplementary file 1 — Additional file 1. Table S1. The list of m5c regulators. Figure S1 41. drugs had lower IC50 values in the high-risk group. Figure S2. The PPI network and GO analysis. (a) The PPI network. (b) Biological process analysis. (c) Cellular component analysis and molecular function analysis. Figure S3. In vitro experiments. (a)AC005562.1 are overexpressed in OC cell lines. (b) siRNA-3 significantly knocked down the AC005562.1 in A2780. (c) siRNA-2 significantly knocked down the AC005562.1 in SKOV3. (d,e) OC cell viability was evaluated with CCK-8 assays at 0, 24, 48, and 72 h post-transfection. *P < 0.05, **P < 0.01, ***P < 0.001. [file 13048_2023_1182_MOESM1_ESM.docx]

Table S1:The list of m5c regulators.

| id | group |
| --- | --- |
| NSUN2 | writers |
| NSUN3 | writers |
| NSUN4 | writers |
| NSUN5 | writers |
| NSUN6 | writers |
| NSUN7 | writers |
| NOP2 | writers |
| TRDMT1 | writers |
| DNMT3A | writers |
| DNMT3B | writers |
| ALYREF | readers |
| TET2 | erasers |
| ALKBH1 | erasers |
| YBX1 | readers |
| TET3 | erasers |
| DNMT1 | writers |


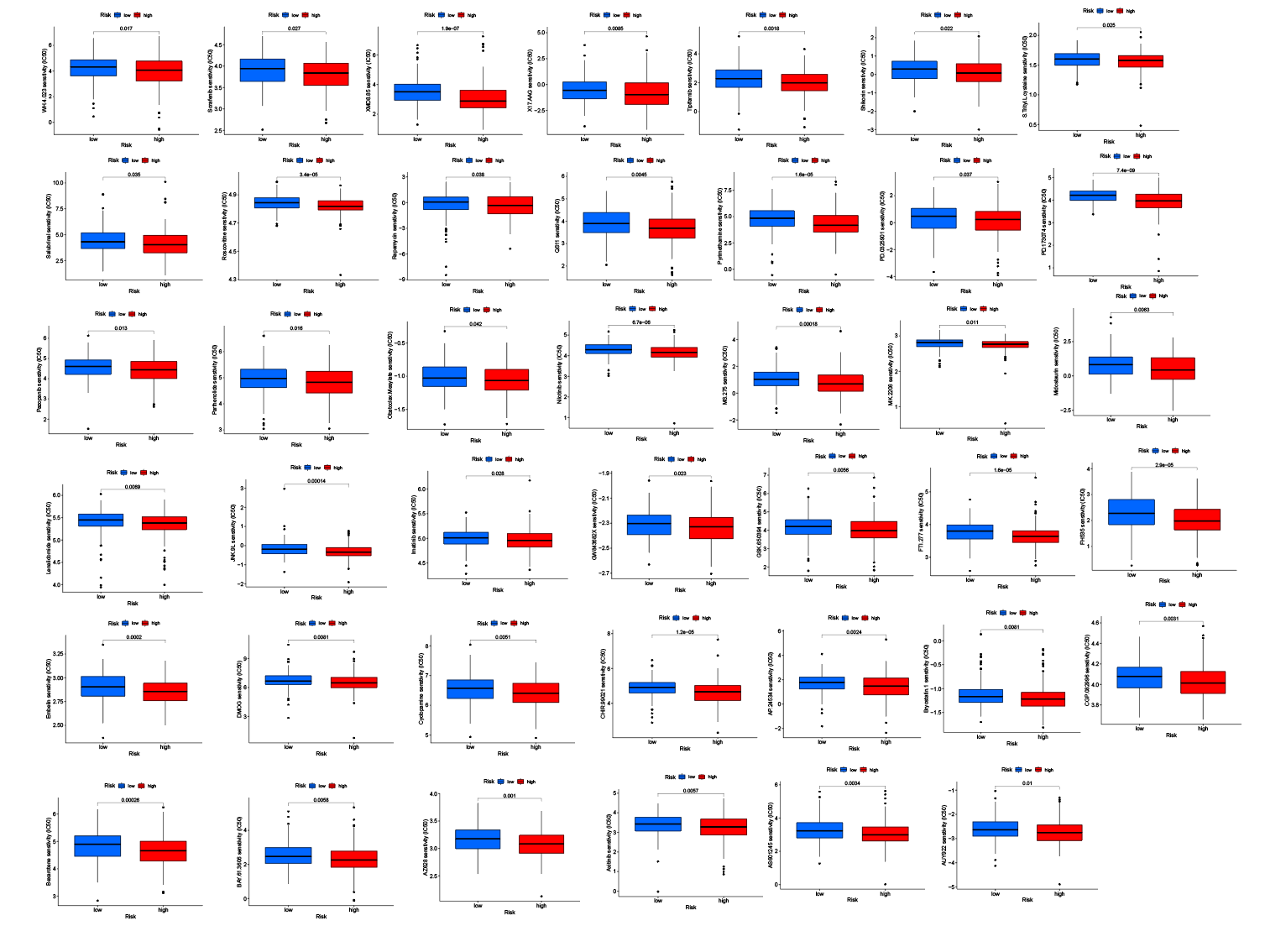


Figure S1 41 drugs had lower IC50 values in the high-risk group.


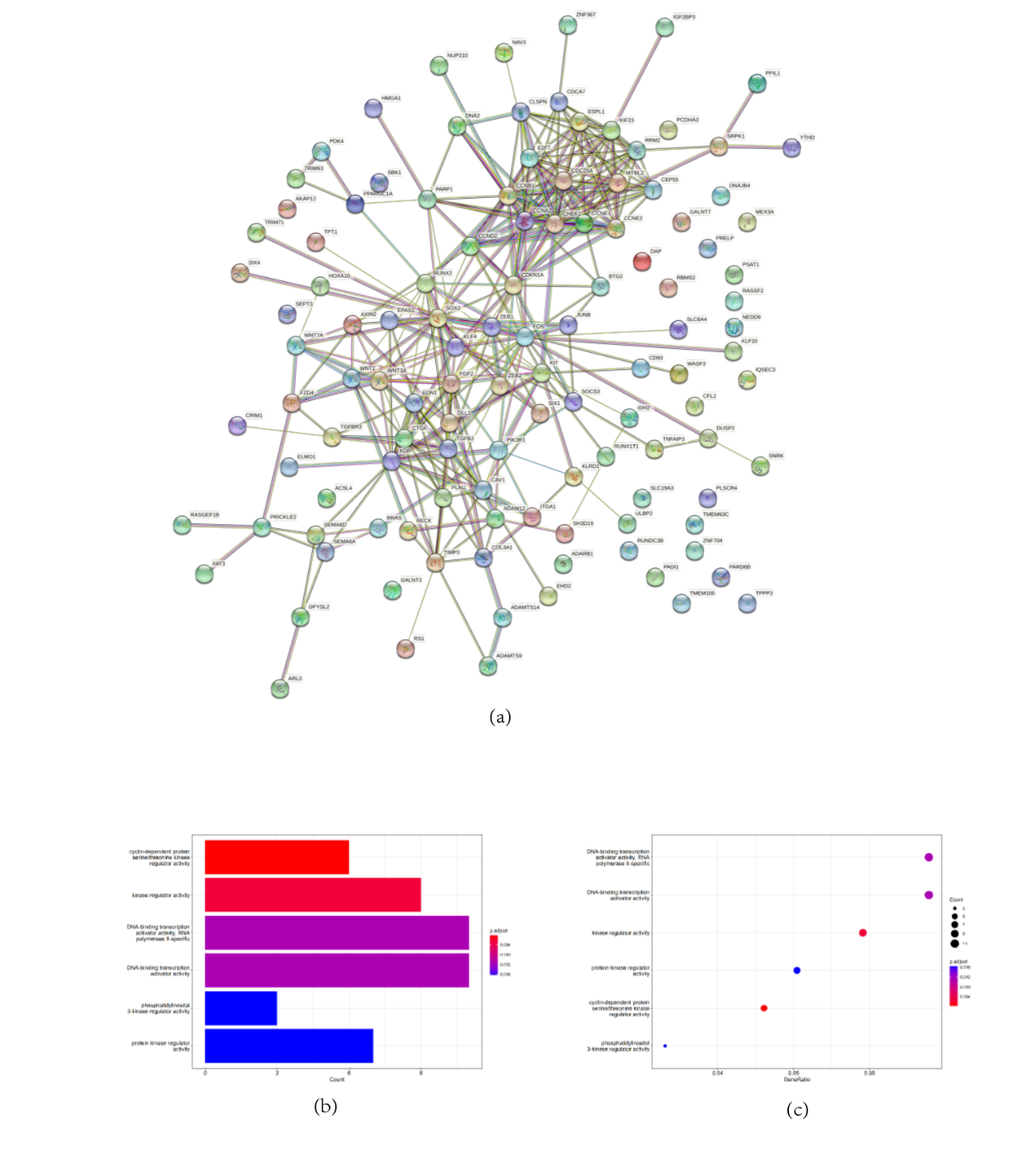


Figure S2 The PPI network and GO analysis. (a) The PPI network. (b) Biological process analysis. (c) Cellular component analysis and molecular function analysis.


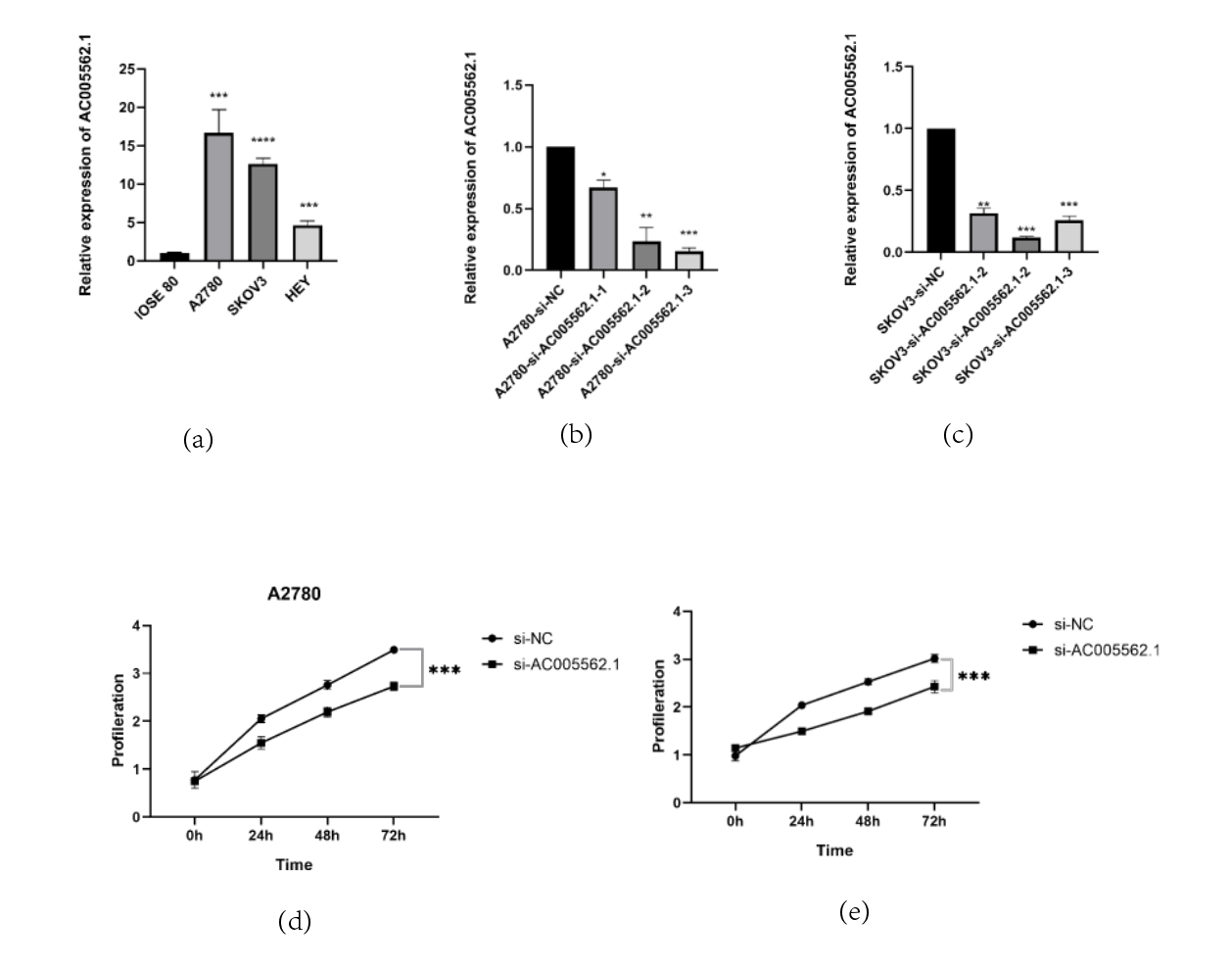


Figure S3 In vitro experiments. (a)AC005562.1 are overexpressed in OC cell lines. (b) siRNA-3 significantly knocked down the AC005562.1 in A2780. (c) siRNA-2 significantly knocked down the AC005562.1 in SKOV3. (d,e) OC cell viability was evaluated with CCK-8 assays at 0, 24, 48, and 72 h post-transfection. *P < 0.05, **P < 0.01, ***P < 0.001.
